# Neuroinflammatory Processes, A1 Astrocyte Activation and Protein Aggregation in the Retina of Alzheimer’s Disease Patients, Possible Biomarkers for Early Diagnosis

**DOI:** 10.3389/fnins.2019.00925

**Published:** 2019-09-04

**Authors:** Alfonso Grimaldi, Natalia Pediconi, Francesca Oieni, Rocco Pizzarelli, Maria Rosito, Maria Giubettini, Tiziana Santini, Cristina Limatola, Giancarlo Ruocco, Davide Ragozzino, Silvia Di Angelantonio

**Affiliations:** ^1^Center for Life Nanoscience, Istituto Italiano di Tecnologia, Rome, Italy; ^2^Department of Physiology and Pharmacology, Sapienza University, Rome, Italy; ^3^Crestoptics S.p.A., Rome, Italy; ^4^IRCCS Neuromed, Pozzilli, Italy; ^5^Department of Physics, Sapienza University, Rome, Italy

**Keywords:** Alzheimer’s disease, microglia, astrocytes, retina, beta-amyloid, tau, human, neurodegeneration

## Abstract

Alzheimer’s disease (AD), a primary cause of dementia in the aging population, is characterized by extracellular amyloid-beta peptides aggregation, intracellular deposits of hyperphosphorylated tau, neurodegeneration and glial activation in the brain. It is commonly thought that the lack of early diagnostic *criteria* is among the main causes of pharmacological therapy and clinical trials failure; therefore, the actual challenge is to define new biomarkers and non-invasive technologies to measure neuropathological changes *in vivo* at pre-symptomatic stages. Recent evidences obtained from human samples and mouse models indicate the possibility to detect protein aggregates and other pathological features in the retina, paving the road for non-invasive rapid detection of AD biomarkers. Here, we report the presence of amyloid beta plaques, tau tangles, neurodegeneration and detrimental astrocyte and microglia activation according to a disease associated microglia phenotype (DAM). Thus, we propose the human retina as a useful site for the detection of cellular and molecular changes associated with Alzheimer’s disease.

## Introduction

Alzheimer’s disease (AD) is a neurodegenerative disorder leading to dementia during elderly. This pathology is manifested with cognitive and psychiatric symptoms such as memory and cognitive impairments, behavioral abnormalities, disorientation and circadian rhythms disturbances (Hart et al., 2016). Due to population aging, AD cases are constantly increasing and it is estimated that by the year 2050 AD will globally affects about 115 million people (Alzheimer’s Disease International World Alzheimer Report, 2009; Scheltens et al., 2016) thus posing a growing concern on public health.

Studies from post-mortem brains revealed that, at the neuropathological level, distinctive features of AD include aggregation of amyloid beta protein (Aβ) and tau protein hyperphosphorylation (pTau), which cause synapses loss and neuronal degeneration. These plaques and tangles are generally associated with activated microglia and reactive astrocytes in AD brain (Ahmed et al., 2017; Henstridge et al., 2019). Over the years several candidate drugs have been proposed as a putative therapy for AD. However, despite many attempts, a definitive cure has not been found yet and only symptomatic treatments are available.

A possible explanation for this arises from the fact that AD is a complex multifactorial disorder, while pharmacological treatments are preferentially directed against a specific cellular target. Moreover, proteins aggregation can occur decades before the appearance of neurological symptoms, thus making interventions at later stages less effective.

It is therefore tempting to speculate that AD diagnosis at earlier stages, possibly when synapses and neuronal functions are not yet compromised, could give better results in terms of pharmacological and clinical intervention (Frisoni et al., 2017).

The search for early biomarkers in AD has become a very attractive area of study. Imaging brain changes in Aβ with techniques such as computed tomography (CT), magnetic resonance index (MRI) and positron emission tomography (PET) in combination with the analysis of cerebrospinal fluid (CSF) have established Aβ and tau as an indicator of the AD disease.

In particular, both PET and CSF biomarkers allow to diagnose AD during the prodromal phase of the disease (Rowe et al., 2013; Petersen et al., 2016), but pitfalls such as high cost and invasiveness make these tests unsuitable for a population-wide screening.

Recently, researchers focused their interests on the retina in order to find new biomarkers for AD. The rationale behind this choice rely on the fact that, the retina and the brain share a common embryological origin therefore they could share pathological mechanisms as well (Sernagor et al., 2001; Kavcic et al., 2011; Chang et al., 2014; Lee et al., 2014; Bambo et al., 2015; Javaid et al., 2016). Recent studies have shown that anatomical alterations such as thinning of the ganglion cell and retinal nerve fiber layers can be detected already during AD early stages (Thomson et al., 2005; Paquet et al., 2007) thus strengthening the idea that the retina could be used in early AD diagnosis. However, far from reaching a consensus, studies on retinal tissues from AD patients and mouse models produced contrasting results. For instance, amyloid plaques have been found in AD *post-mortem* retinas (but not in control cases) by Löffler et al. (1995). Similar results have been confirmed by other research groups (Hoh Kam et al., 2010; Alexandrov et al., 2011; Koronyo-Hamaoui et al., 2011; Koronyo et al., 2017), in addition a positive correlation between inclusion body and cortical amyloid burden has been observed (Snyder et al., 2016). On the contrary, den Haan and collegues detected a signal for phosphorilated Tau (pTau) in the inner and outer plexiform layer of the retina (IPL and OPL respectively), but they did not find any signal for Aβ plaques and neurofibrilary tangles (den Haan et al., 2018). Most likely, these discrepancies may be attributed to differences in staining methods, sections preparation and time of tissue harvesting. Moreover, restricting retinal analysis to the presence of protein aggregates and vascular alterations solely may be poorly specific for AD diagnosis, as Aβ deposits have been found also in other pathological conditions such as macular degeneration (Zhao et al., 2015). Beyond classical neuropathological hallmarks, proteomic analysis of cerebrospinal fluid from AD patients revealed the upregulation of proteins related to microglia and astrocytes activation. For instance, Aβ plaques aggregation can activate astrocytes and microglia thus inducing the release of mediators of inflammation such as interleukin-1 beta (IL-1β) and ultimately cellular and neuronal apoptosis both in brain and retina (Liu et al., 2014).

In neurodegenerative disease, microglia response consists in migrating to sites of damage or injury, secreting numerous inflammatory molecules, and phagocytizing debris and aggregated proteins (Heneka et al., 2015; Song and Colonna, 2018). However, microglia, astrocytes and immune signaling are not just secondary players in disease processes but actually contribute to synaptic and neuron loss and buildup of pathogenic proteins even at the earliest stages of disease (Hong et al., 2016; Shi et al., 2017; Sosna et al., 2018). Aggregation of Aβ plaques can lead to inflammatory reactions, astrocytes and microglia activation, release of inflammatory cytokines such as interleukin-1 beta (IL-1β) and cellular and neuronal apoptosis in both the brain and retina (Liu et al., 2014). Moreover, the misregulated expression of glia released soluble factors in AD has been linked to disease associated microglia, and to microglia induced NLRP3 inflammasome and complement activation (Heneka et al., 2013; Sheedy et al., 2013; Lian et al., 2015; Iram et al., 2016) and synaptic loss (Hong et al., 2016). Moreover, a specific microglia phenotype has been described in neurodegenerative diseases characterized by the downregulation of microglia homeostatic genes, the upregulation of specific degeneration associated markers (DAM), and sustained by the upregulation of TREM2 (Yeh et al., 2017; Deczkowska et al., 2018).

To gain a better understanding and to identify new molecular targets for a more complete panel of putative biomarkers for AD diagnosis, we performed immunostaining on human retinas obtained from both AD patients and age matched controls. We report the presence of Aβ and pTau protein aggregates together with neuronal loss. We also report, for the first time, the upregulation of IL1-β on microglia and the presence of neurotoxic A1 astrocytes in AD retina. These findings highlight protein aggregates and cellular markers as targets to be considered for AD diagnosis.

## Materials and Methods

### Human Samples

Human retinal slices from AD patients and age matched controls were purchased from Human Eye Biobank for Research, St Michael Hospital, Toronto, Canada. Prior to death, donors signed informed consent for autopsy and use of tissue and medical records for research purposes. No documented history of eye disease was reported for the AD or control cases. The use of human tissue has been approved by the Ethics Committee of Fondazione Santa Lucia I.R.C.C.S. to GR.

### Immunofluorescence

Slices were deparaffined with absolute Xylene (3 × 5 min) and rehydrated by scaling down from 100% to 50% ethanol, 2 × 10 min for each concentration. After PBS washes (3 × 5 min), antigen retrieval has been performed by soaking slices for 40 min in a warm solution containing (in mM): 10 Na-citrate, 0.05% Tween 20, pH 6.0, 90°C. Subsequently, slices were incubated for 45 min in a blocking solution (3% goat serum and 0.3% Triton X-100 in PBS). Primary antibodies diluted in blocking solution were incubated over-night at 4° [anti-βTubulin III, T2200, Sigma, clone Aa441-450, 1:500; anti-GFAP, MAB360, Millipore, clone GA5; 1:400; anti-Iba1, 019-19741, Wako, 1:400; anti-OPN, 691302, Biolegend, clone A15059B, 1:100; Anti-βAmyloid, 8243s, CELL SIGNALING, clone D54D2, 1:100; anti-Cleaved-Casapase3, 9661s, CELL SIGNALING, clone Asp175, 1:100; anti-PhosphoTau (Thr212, Ser214), MN106, INVITROGEN clone AT100 1:50; anti-PhosphoTau (Ser 202, Thr205), INVITROGEN, clone AT8, 1:40; anti-C3d, A0063, Dako, 1:500; anti-IL-1β, sc-32294, Santa Cruz Biotechnology, clone E7-2-hIL1β, 1:50]. After three washes in PBS, sections were stained for 45 min with a secondary antibody and Hoechst in order to visualize nuclei, then coverslips were mounted with Diamond Antifade Mountant (Molecular Probes) and images acquired with a confocal microscopy.

### Confocal Spinning Disk and VCS Microscopy

The acquisition of the images was performed through a Nikon Eclipse Ti equipped with a X-Light V2 spinning disk combined with a VCS (Video Confocal Super resolution) module (CrestOptics) based on structured illumination and with a LDI laser source (89 North). The images were acquired by using Metamorph software version 7.10.2. (Molecular Devices) with a 60x PlanApo λ oil objective (1.4 numerical aperture) and sectioning the slice in Z with a step size of 0.2 μm for spinning disk and 0.15 μm for VCS to obtain a total Z-stack of about 10 μm. In order to achieve super-resolution, raw data obtained by the VCS module have been processed with a modified version of the joint Richardson-Lucy (jRL) algorithm (Ingaramo et al., 2014; Ströhl and Kaminski, 2015; Chakrova et al., 2016), where the out of focus contribution of the signal has been explicitly added in the image formation model used in the jRL algorithm, and evaluated as a pixel-wise linear “scaled subtraction” (Heintzmann and Benedetti, 2006) of the raw signal. The acquisitions obtained were transformed into a z-projection and then analyzed using the ImageJ software.

### Laser Scanning Acquisitions

For conventional confocal laser scanning analysis of retinal slices, images acquisition has been performed through a confocal laser scanning microscope (microscope (FV10i Olympus) equipped with a 60x water immersion objective. Acquired images were processed and analyzed off line using ImageJ. For each retina six images were acquired. For every image the maximum intensity projections of z-series stacks was created.

### Microglia Density Analysis

The number of Iba1^+^ microglia cells has been reported as number of somas per acquired area (400 μm^2^). To quantify microglia density, the same images were analyzed by Metamorph software. A z-projection based on the maximal intensity signal was obtained and after threshold setting, fluorescence intensity value has been recorded. Data are expressed as area occupied by fluorescent cells *versus* total slice area.

### Astrogliosis Analysis

Astrogliosis analysis has been performed by staining retinas section for GFAP and C3. Following threshold adjustment astrogliosis was quantified as fluorescence intensity above threshold. Data are expressed as area occupied by fluorescent cells versus total slice area. For the measurement of GFAP/C3 colocalization, the value of GFAP-overlapping the C3 signal was considered.

### Analysis of Neurodegeneration

Confocal images of cells positive for cleaved caspase 3, an effector enzyme of the apoptotic pathway, were analyzed by means of Metamorph software. We manually counted the number of cells positive for the signal of the cleaved caspase 3 within each image. Thereafter, this number has been divided by the total number of ganglion neurons counted in the region of interest (ROI) and thus expressed as percentage of degenerating ganglion cells in each slice.

### FISH

Sections were deparaffined with absolute Xylene (3 × 5 min) and partially rehydrated with 70% ethanol (2 × 10 min each) before proceeding with RNA FISH staining. RNA *in situ* hybridization was performed as described previously (Raj and Tyagi, 2010) with minor modifications: briefly, sections were incubated in Wash Buffer A (Stellaris, Biosearch Technologies) for 5 min and then, in the Hybridization Buffer supplemented with 2 mM VRC complex (Sigma, R3380) and 125 nM TREM2 FISH probes 3′-end labeled with Quasar 670 fluorophore (Biosearch Technologies, SMF-1063-5). Incubation was performed overnight at 37°C in Top Brite automatic slide hybridizer (Resnova). After two washes in Wash buffer A for 30 min and one wash with Wash Buffer B for 5 min at 37°C, Hoechst was added for 15 min at room temperature. Finally, slices were mounted with ProLong Diamond Antifade Mountant (Thermo Fischer Scientific P-m36961) and analyzed with a confocal laser scanning microscope. Immunofluorescence for IBA1 was performed sequentially to RNA FISH staining.

### Protein Aggregates

The size of the amyloid plaques and tau tangles was studied on images acquired with the confocal microscope and subsequently analyzed with the Metamorph program (version 7.6.5.0). The “Trace Region” function of this program makes it possible to surround the deposits of these proteins and to obtain their volume expressed in μm^3^. Their density has been studied with the ImageJ program through which it was possible to count the number of these aggregates within each slice analyzed.

### Statistics and Data Analysis

Data are shown as the Mean ± SEM. Statistical significance between controls and AD patients was assessed with the non-parametric Mann–Whitney test or *T*-test as indicated. A *p*-value < 0.05 was considered significant. All statistical analyses were done using Sigma Plot 11.0, Origin or Clampfit software.

## Results

### Typical Aβ and pTau Protein Aggregates in AD Human Retina

The accumulation of Aβ and pTau aggregates in the retinal tissue has been reported on both AD human tissue (Koronyo-Hamaoui et al., 2011; Koronyo et al., 2017) and mouse models (Criscuolo et al., 2018; Grimaldi et al., 2018), thus suggesting a putative use of the eye as a valuable structure for the study and the diagnosis of AD and other neurodegenerative disease. However, due to the complex nature of the disease, and to the positive Aβ staining in macular degeneration (Lynn et al., 2017), a more comprehensive panel of biomarkers needs to be defined to avoid misleading AD diagnosis. Using human retinal slices from AD patients and controls (samples were obtained from the St. Michael’s Hospital Human Eye Biobank), we first confirmed the presence of Aβ and pTau aggregates in the retinal layers.

**FIGURE 1:**
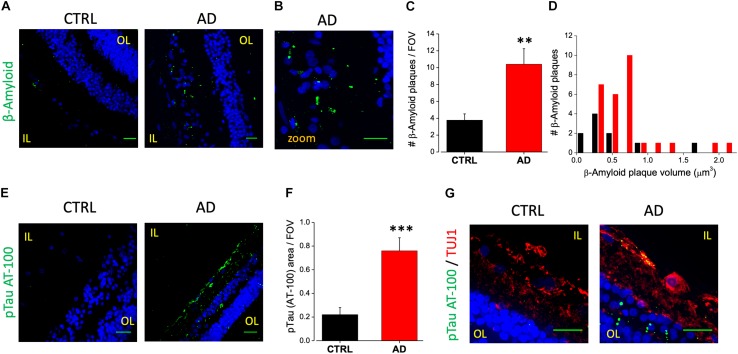
Human AD retina displays β-amyloid and pTau aggregates. **(A)** Representative images of retinal slices from AD patients and control cases immunolabeled with anti-β-amyloid antibody (green) and Hoechst for nuclei visualization (blue); bar 20 μm. **(B)** Insert representing β-amyloid staining in AD retina at higher magnification; bar 20 μm **(C)** Number of β-amyloid plaques/field of view (^∗∗^*p* < 0.01 AD vs. Ctrl; *t*-test; *n* = 45/5 fields/patients). **(D)** Distribution of β-amyloid plaque volume measured in AD patients and control cases. **(E)** Representative images of retinal slices from AD patients and control cases immunolabeled with anti-p-tau AT-100 antibody (green) and Hoechst for nuclei visualization (blue); bar 20 μm. **(F)** Quantification of p-tau AT-100 area covered by fluorescent signal/field of view (^∗∗∗^*p* < 0.001 AD vs. Ctrl; *t*-test; *n* = 38/6 fields/patients). **(G)** Representative images of retinal slices from AD patients and control immunolabeled with anti-p-tau AT-100 (green) and anti-TUJ1 as RGC marker (red) at higher magnification (Hoechst for nuclei visualization in blue; bar 20 μm). IL, inner layer; OL, outer layer.

We analyzed retinal cross sections from 10 clinically and neuropathologically confirmed AD patients (mean age ± SEM: 85.7 ± 2.7 years; range 71–98 years; 8 females and 2 males) and 10 healthy controls (mean age ± SEM: 75.3 ± 2.9 years; range: 65–93 years; 6 females and 4 male). In all AD patients we detected retinal Aβ immunoreactivity and deposits both in the inner and outer layers (minimum diameter: 110 nm; Figures 1A,B). Aβ staining, evaluated as Aβ plaques number in a region of 400 μm^2^, was significantly higher in AD patients (10.4 ± 1.8 *n* = 45/5 fields/patients) compared to controls (3.8 ± 0.7 *n* = 44/5 fields/controls; *p* < 0.01; Figure 1C). Sparse and diffuse retinal Aβ deposits were found occasionally also in control slices (Figure 1A, left), as previously reported (Koronyo et al., 2017). Plaques volume measured on 3D stacks was higher in AD patients respect to controls as reported in the histogram in Figure 1D.

In order to assess the presence of hyperphosphorylated tau isoforms we performed immunofluorescence analysis using two different monoclonal antibodies directed against the hyperphosphorylated tau protein (pTau; clone AT-8 and clone AT-100, Figure 1E). Following the staining with the AT-100 clone we observed a strong and diffuse staining for pTau both in the IPL and OPL in the majority of AD patients (6/10). Indeed, pTau immunoreactivity was significantly higher in the AD retina compared to age matched controls as quantified by fluorescence intensity in each field of view (AD: 0.76 ± 0.11, *n* = 38/6 fields/patients; CTRL: 0.22 ± 0.06, *n* = 32/6 fields/controls; *p* < 0.005; FOV = 400 μm^2^; Figure 1F), thus confirming the accumulation of neurofibrillary tangles deposits in AD patients retina (Figure 1E, right). Using the AT-8 clone we found discrete pTau immunofluorescence in the IPL and in the OPL only in two out of 10 patients analyzed; in these AD patients the number of pTau tangles was significantly higher respect to control (AD: 4.4 ± 1.7, *n* = 15/2 fields/patients; CTRL: 0.9 ± 0.4; *n* = 15/2 fields/controls; *p* < 0.05; not shown). Co-labeling the retina with AT-100 and TUJ1 as neuronal marker indicated that in AD retina pTau expression was mainly in the Retinal Ganglion Cells (RGC) of the inner layer (IL; Figure 1G).

These findings clearly demonstrate an increase in Aβ and pTau aggregates in the retina of AD patients respect to controls thus pointing at them as putative biomarkers for AD diagnosis.

### AD Human Retina Displays Neurodegeneration in the Ganglion Cell Layer

**FIGURE 2:**
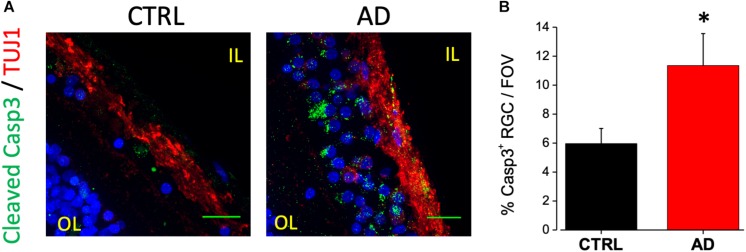
Ganglion cell neuron degeneration in AD patient’s retina. **(A)** Representative images of retinal slices from AD patients and control cases immunolabeled with anti-cleaved caspase-3 antibody (green), anti-TUJ1 as RGC marker (red) and Hoechst for nuclei visualization (blue); bar 20 μm. **(B)** Bar chart representing the percentage of RGC neuron positive for cleaved caspase-3 on each field of view (^∗^*p* < 0.05 AD vs. Ctrl; *t*-test; *n* = 17/4 fields/patients). IL, inner layer; OL, outer layer.

An increased cleavage of proteins such as APP and presenilins operated by caspase-3, has been associated with neurodegeneration in AD (Louneva et al., 2008). In a previous paper from our laboratory, we reported immunoreactivity for the cleaved caspase-3 at the level of the RGC layer of the 3xTg-AD mouse model (Grimaldi et al., 2018). A positive staining for caspase-3 is also present in the IL of human retinas (Figure 2A, green dots) in TUJ1 positive cells (red). In particular we found that the number of caspase-3 positive retinal ganglion cells in each field of view (FOV) examined was increased compared to age matched controls (AD: 11.4 ± 2.2%, *n* = 17/6 fields/patients; CTRL: 6.0 ± 1.0%, *n* = 18/6 fields/control; *p* < 0.05; Figure 2B) These results indicated that the AD retinal IL is more subject to neuronal death thus suggesting that visual defects and optic nerve thinning observed in AD may rely on retinal ganglion cells neurodegeneration.

### Increased Astrocytosis and Microglia Reactivity in AD Patients Retina

**FIGURE 3:**
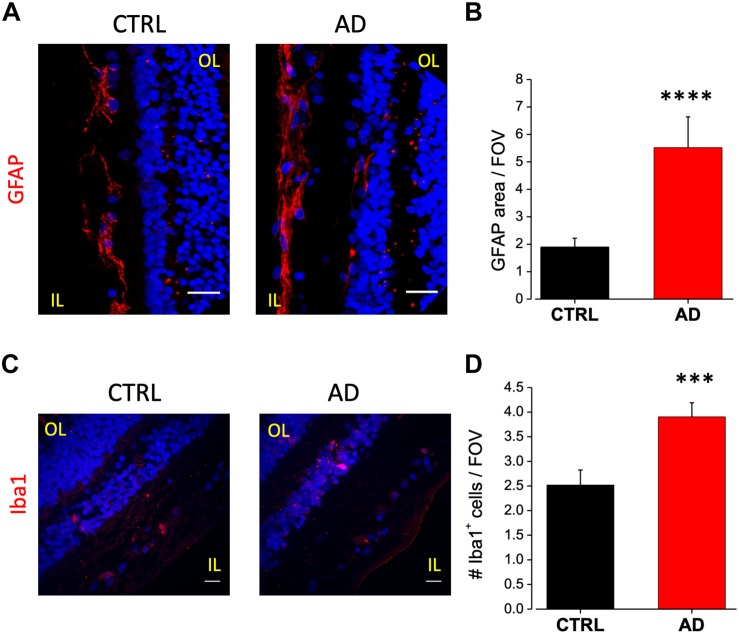
Increased astrocytes and microglia cell density in the retina of AD patients. **(A)** Representative images of retinal slices from AD patients and control cases immunolabeled with anti-GFAP antibody (red) and Hoechst for nuclei visualization (blue); bar 20 μm. **(B)** Quantification of GFAP area covered by fluorescent signal/field of view (^∗∗∗∗^*p* < 0.001 AD vs. Ctrl; *t*-test; *n* = 44/6 fields/patients). **(C)** Representative images of retinal slices from AD patients and control cases immunolabeled with anti-Iba1 antibody (red) and Hoechst for nuclei visualization (blue); bar 20 μm. **(D)** Quantification of Iba1 area covered by fluorescent signal/field of view (^∗∗∗^*p* < 0.005 AD vs. Ctrl; *t*-test; *n* = 73/10 fields/patients). IL, inner layer; OL, outer layer.

In AD brain, astrocytes have been found closely associated with fibrillar amyloid plaques suggesting that Aβ accumulation may serves as a cue for the activation of this cell type (Henstridge et al., 2019). Astrocytes activation can have a neuroprotective effect but it may trigger the release of pro-inflammatory species such as cytokines and chemokines (Stadelmann et al., 2002; Farina et al., 2007). To evaluate putative astrogliosis in the retina of AD patients we performed immunostaining for the GFAP. Analysis of acquired images showed a marked astrogliosis localized at the level of the ganglion cell layer. Astrogliosis may arise also as a consequence of aging however, the amount of astrocyte activation was more pronounced in the AD retina compared to controls (Figure 3A), as quantified by fluorescence intensity in each field of view (AD: 5.5 ± 1.1, *n* = 44/6 fields/patients; CTRL: 1.9 ± 0.3, *n* = 44/6 fields/controls; *p* < 0.001; Figure 3B).

As for astrocytes, also microglia activation can show a dual role in inflammatory processes. Increased microglia reactivity in the retina has been observed in post-mortem tissues from AD patients (Beach et al., 1989) and in the 3xTg-AD mouse model (Grimaldi et al., 2018). Immunostaining against Iba1 a microglia marker, revealed that this cell type was mainly present in two layers: the inner plexiform and the outer plexiform layers (Figure 3C). Microglia cell density was increased in AD patients retina compared to age matched controls (AD: 3.9 ± 0.3; *n* = 73/10 fields/patients; CTRL: 2.5 ± 0.3, *n* = 73/10 fields/controls; *p* < 0.005; Figure 3D).

These results, confirming the presence of altered astrocyte and microglia density in human AD retina, suggest the possibility to add markers for glial activation in the set of retinal biomarkers for AD diagnosis.

### AD Human Retina Displays Upregulation of Neurotoxic Microglia and Astrocytes

Several different proteins have been demonstrated to be involved in the interplay among neurons, astrocytes and microglia in neurodegenerative diseases. Particularly, altered expression of these proteins in AD has been linked to disease associated microglia, to microglia induced detrimental astrocytes activation (Heneka et al., 2013; Sheedy et al., 2013) and synaptic loss (Hong et al., 2016). Here we examined and compared the expression of IL-1β, TREM-2 (triggering receptor expressed on myeloid cells-2), complement component C3 and OPN (Osteopontin) between AD and control samples.

**FIGURE 4:**
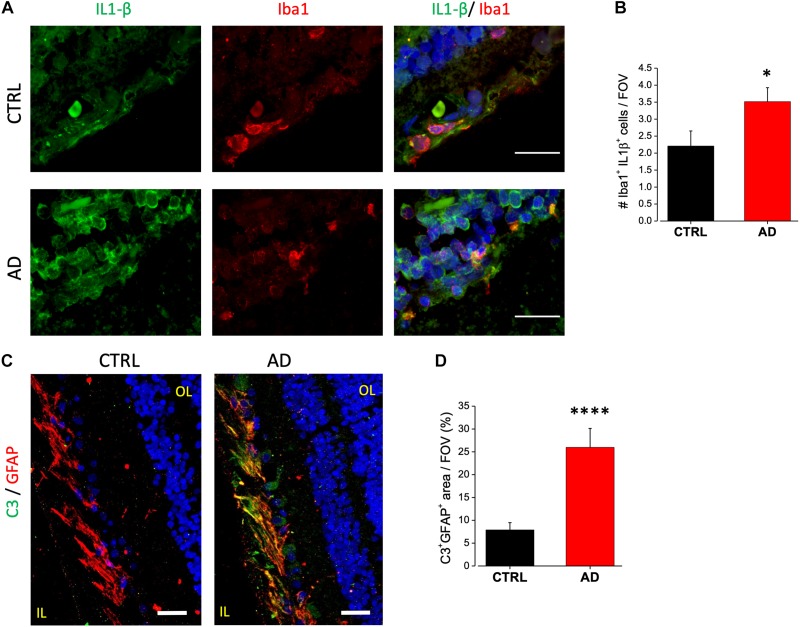
AD patient’s retina display complement C3 and IL-1β upregulation. **(A)** Representative images of retinal slices from AD patients (bottom) and control cases (top) immunolabeled with anti-IL-1β antibody (green), Iba1 (red) and Hoechst for nuclei visualization (blue); bar 20 μm. **(B)** Quantification of cells positive for both Iba1 and IL-1β signal/field of view (^∗^*p* < 0.05 AD vs. Ctrl; *t*-test; *n* = 31/5 fields/patients). **(C)** Representative images of retinal slices from AD patients and control cases immunolabeled with anti-GFAP antibody (red), C3 (green) and Hoechst for nuclei visualization (blue); bar 20 μm. **(D)** Quantification of GFAP/C3 co-localization area/field of view (^∗∗∗∗^*p* < 0.001 AD vs. Ctrl; *t*-test; *n* = 45/6 fields/patients). IL, inner layer; OL, outer layer.

Immunofluorescence analysis of IL-1β expression on AD retinal slices showed enhanced IL-1β cytoplasmic staining in the inner nuclear layer (INL) of AD patients compared to age matched controls (Figure 4A, left). IL-1β immunostaining in AD patients retina was found to be colocalized with Iba1-expressing cells, a marker of microglia, but not with the astrocytes marker GFAP (not shown). Indeed, co-labeling with Iba1 (Figure 4A, right) revealed that the number of microglia cells positive for IL-1β was significantly higher compared to control (AD: 3.5 ± 0.4, *n* = 31/5 fields/patients; CTRL: 2.2 ± 0.4, *n* = 29/5 fields/controls; *p* < 0.05; Figure 4B; average number of Iba1/IL-1β + cells for each FOV), with almost all microglia cells expressing IL-1β in AD.

Astrocytic C3 upregulation has been demonstrated to be downstream of the microglia induced IL1-β activation. Moreover, its expression seems to be regulated by β-amyloid in both AD mouse models and human brain (Lian et al., 2015). C3 upregulation is now considered to be a marker for detrimental A1 astrocytic phenotype taking part in AD related synaptic loss. We examined C3 expression in human retina by immunofluorescence analysis. We found C3 to be expressed in the ganglion cell layer. Co-staining with GFAP revealed that C3 immunoreactivity was confined in GFAP positive cells as shown in Figure 4C. Fluorescence intensity quantification showed that the percentage of C3 positive astrocytes was strongly upregulated in AD retina respect to control (AD: 26 ± 4, *n* = 45/6 fields/patients; CTRL: 7.8 ± 1.6, *n* = 46/6 fields/controls; *p* < 0.001; Figure 4D).

**FIGURE 5:**
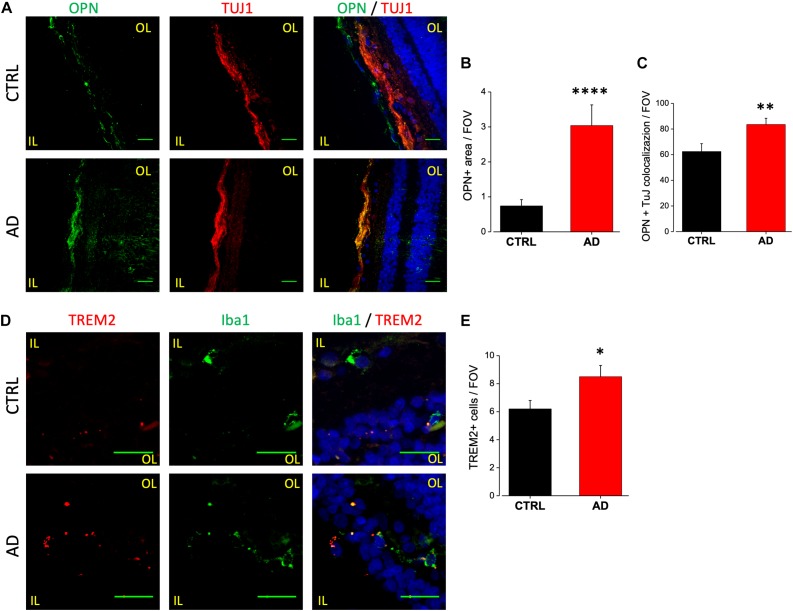
Osteopontin and TREM2 expression in AD patient’s retina. **(A)** Representative images of retinal slices from AD patients (bottom) and control cases (top) immunolabeled with anti-OPN (green), tuj-1 (red) and Hoechst for nuclei visualization (blue); bar 20 μm. **(B)** Quantification of OPN area covered by fluorescent signal/field of view (^∗∗∗∗^*p* < 0.001 AD vs. Ctrl; *t*-test; *n* = 24/3 fields/patients). **(C)** Quantification of OPN/TUJ1 co-localization area/field of view, in% (^∗∗^*p* < 0.01 AD vs. Ctrl; *t*-test; *n* = 36/6 fields/patients). **(D)** Representative images of retinal slices from AD patients (bottom) and control cases (top) stained with anti TREM2 mRNA fluorescent probe (red), anti-Iba1 (green) and Hoechst for nuclei visualization (blue); bar 20 μm. Note that cells expressing TREM2 are not always positive for Iba1. **(E)** Number of TREM2 positive cells/field of view (^∗^*p* < 0.05 AD vs. Ctrl; *t*-test; *n* = 31/4 fields/patients). IL, inner layer; OL, outer layer.

Upregulation of OPN in AD patients CSF may arise from neurons, microglia or both. Indeed, while the OPN encoding gene SPP1 (secreted phosphoprotein 1) is now considered a good DAM microglia marker (Ulland et al., 2017), AD brains display OPN upregulation in CA1 pyramidal neurons (Wung et al., 2007). We analyzed the expression level of OPN in human retinal slices of AD patients and age matched controls by immunofluorescence analysis using a monoclonal antibody against the full length OPN peptide (Figure 5A, left). We found significant upregulation of OPN expression in AD retinas, quantified as fluorescence intensity over threshold in each field of view (400 μm^2^) (AD: 3.0 ± 0.6, *n* = 24/3 fields/patients) compared to age matched controls (CTRL: 0.7 ± 0.2, *n* = 27/4 fields/patients; *p* < 0.001) (Figure 5B). OPN expression was localized in the retinal ganglion cell layer as demonstrated by the co-staining against tubulin isoform βIII (TUJ1; Figure 5A, middle), a selective marker for RGC neurons. Indeed, the percentage of RGC neurons positive for OPN (Figure 5A, left) was significantly higher in AD respect to control retinas (AD: 84 ± 5%, *n* = 36/6 fields/patients; CTRL: 63 ± 6%, *n* = 34/6 fields/patients; *p* < 0.01; Figure 5C). Conversely, co-labeling with anti-Iba1 did not show upregulated OPN expression on microglia (data not shown).

TREM2 is now considered a crucial regulator in promoting microglia responses to Aβ in AD. TREM2 transcript has been shown to be over-expressed by microglia both in the brain of AD mouse models as well as human patients (Frank et al., 2008; Lue et al., 2015; Wang et al., 2015), suggesting that TREM2 up-regulation could mirror with AD progression. Moreover, in the 3xTg-AD mouse model, we reported the upregulation of TREM2 mRNA in sorted retinal microglia cells (Grimaldi et al., 2018). Due to the lack of specific antibodies against human TREM2, FISH experiments have been performed in order to evaluate the expression level of TREM2 transcript in retinal slices of AD patients and age matched controls (Figure 5D). The analysis of TREM2 RNA levels obtained with fluorescence threshold analysis did not show, on average, any difference between AD and control retinas (AD: 0.18 ± 0.03, *n* = 31/4 fields/patients; CTRL: 0.22 ± 0.06, *n* = 37/4 fields/controls; *p* = 0.3; data not shown). However, the number of TREM2 positive cells in each field of view (400 μm^2^) was higher in AD retina respect to control (AD: 8.5 ± 0.8, *n* = 31/4 fields/patients; CTRL: 6.2 ± 0.6, *n* = 37/4 fields/controls; *p* < 0.05; Figure 5E), indicating an increased TREM-2 expression. When TREM2 FISH analysis was performed together with immunofluorescence for Iba1, we observed Iba1 positive cells expressing TREM2 in AD retina (Figure 5D, left). It should be noticed, however, that in our experiments TREM2 staining was not exclusively confined to Iba1-positive cells. This could be ascribed to protein denaturation taking place during FISH protocol, preventing an absolute quantification of TREM2 expressing retinal microglia cells.

These data indicate that detrimental astrocytic and microglia activation can be detected in AD patients retina with increased release of pro-inflammatory compounds that could be considered as possible biomarkers for AD diagnosis.

## Discussion

As of now, a definitive AD diagnosis is made possible only after post-mortem examination of brain tissue (Elahi and Miller, 2017). Despite many attempts there is an urgent need to find new easy-to-acquire, cost-effective strategies for AD diagnosis, even at early stages in order to allow a timely and effective therapeutic program (Frisoni et al., 2017).

The retinal tissue is now considered as a very promising structure to be used in searching of new AD biomarkers as it is thought that it could mirror the pathological changes happening in the brain during the disease progression. Even more, the retina is an accessible structure and this anatomical feature could be of extreme importance in the development of new imaging methods for its examination.

However, Aβ and pTau protein aggregates could not be considered specific markers to AD therefore, a more comprehensive panel of biomarkers is needed.

For this reason, we used *post-mortem* retinal slices from AD patients to investigate the presence of classical AD features and the expression of specific neuron-to-glia signaling proteins found both in the CSF of AD patients and in the brain of AD mouse models (Doens and Fernández, 2014). We here report that AD patients retina show, in addition to the presence of Aβ plaques and pTau tangles, ganglion neuron degeneration, astrogliosis, microglia activation, and up-regulation of specific disease associated neuron-to-glia signaling proteins, such as IL-1β, C3, OPN and TREM2.

In agreement with previous results (Koronyo et al., 2017; den Haan et al., 2018), we observed increased Aβ deposits and diffuse spreading of pTAu signals in the inner retinal layer of AD patients compared to age-matched controls. However, it has to be noticed that only a subset of AD retinal slices here analyzed were positive for pTau staining with AT-8 and AT-100 antibodies, this could be ascribed to pTau specific topographic distribution in human retina; indeed pTau staining was clearly reported in the anterior part of the superior retina (Koronyo et al., 2017; den Haan et al., 2018), and using transverse retinal slices obtained from the Human Eye Bank we could not asses which part of the retina we were analyzing. Due to this technical issue it is plausible that we are underestimating the amount of pTau in the AD patients retina. Also, we show that neuronal apoptosis is significantly higher in the retina of AD patients compared to age matched controls, predominantly in RGC layer.

As it is well known that glial cells are activated in AD associated inflammatory states, we performed immunostaining to evaluate putative difference between controls and AD samples. Although GFAP and Iba1 reactivity was present both in AD and aged controls, the area of GFAP positive staining and the number of microglia cells were significantly higher in AD retinas thus indicating that glia (both astrocytes and microglia) activation is significantly more pronounced in AD retinas compared to controls.

We show here for the first time that retinal microglia of AD patients display, respect to their age matched controls, higher expression of IL-1β a typical marker of pro-inflammatory and DAM microglia (Keren-Shaul et al., 2017) suggesting microglia response to Aβ and pTau accumulation. These results are in line with the increased expression of IL-1β found in brain microglia in human patients and mouse models (as reviewed in Shaftel et al., 2008). Conversely, while in the brain of 5xFAD mouse model IL-1β was overexpressed by astrocytes (Rosenzweig et al., 2019), in human AD and control retina we did not observe IL-1β expression in GFAP positive cells. It should be noted that we found enhanced IL-1β staining also in other cells in the INL of AD retina, probably due to the NLRP3 inflammasome activation and monocytes infiltration.

We also report an increase of TREM2 mRNA level in the retina of AD patients respect to aged controls. The importance of TREM2 in the central nervous system (CNS) is widely recognized, as TREM2 mutations are linked to an increased risk of developing several neurodegenerative diseases (Ulland and Colonna, 2018), and TREM2 RNA has been found to be upregulated in the brain of patients and mouse models (Keren-Shaul et al., 2017). Moreover, we previously reported TREM2 upregulation in retinal microglia sorted from early symptomatic 3xTg-AD mice (Grimaldi et al., 2018). However, while it is evident that TREM2 expression on microglia cells plays a prominent role in driving microgliosis in the brain of AD mouse models and patients (Keren-Shaul et al., 2017), we did not find increased expression of TREM2 transcript on Iba1 positive cells in the retina of AD patients. It can be speculated that in our samples the expression of TREM2 on microglia cells is underestimated due to technical reasons. Indeed, RNA FISH experiments on post-mortem paraffin-embedded tissue is *per se* technically challenging, requiring the use of formamide to increase the hybridization efficiency (Cattoretti et al., 1993; Gilbert et al., 2007). This procedure might induce protein denaturation causing the failure of subsequent immunofluorescence analysis. Another possible explanation may rely on the age of AD patients here analyzed. Indeed, while microglia TREM2 expression is important in triggering and sustaining the DAM phenotype in response to protein aggregates during disease progression (Song and Colonna, 2018; Ulland and Colonna, 2018), downregulation of TREM2 has been reported in human post-mortem retinal tissues of patients suffering from Age-Related Macular Degeneration (Bhattacharjee et al., 2016) where Aβ deposits are present. In this framework it is likely that post mortem retinal tissue from aged patients is not the ideal specimen for TREM2 analysis. Moreover, the observed increase in TREM2 transcript level could be due to infiltrating monocytes negative for Iba1, as reported in the brain of AD patients (Fahrenhold et al., 2018).

The analysis of osteopontin expression in retinal tissue revealed increased OPN staining in AD retinal ganglion cells with evident colocalization with tubulin β-III. This result is partially in contrast with previous findings in the AD brain. Indeed, although OPN transcript (SPP1) expression has been shown to be upregulated in microglia in AD models (Ulland et al., 2017) and thus considered a marker for DAM phenotype, we did not find OPN upregulation on Iba1 positive cells. On the other hand our result is in line with data reporting that the protein osteopontin is upregulated on CA1 hippocampal pyramidal neurons in human AD brain (Wung et al., 2007). Moreover, in mouse retina, OPN is reported to be expressed on RGC (Ju et al., 2000; Duan et al., 2015). These considerations make OPN a good candidate biomarker for retinal AD diagnosis despite its cellular localization.

Here, for the first time we report the presence of A1 astrocytic phenotype in the retina of AD patients, as revealed by the strong upregulation of C3 protein on retinal astrocytes. This result is consistent with the reported presence of A1 reactive brain astrocytes in neurodegenerative diseases (Liddelow et al., 2017). The importance of reactive A1 astrocytes in neurodegenerative diseases rely on the evidence that synaptic loss can be favored by element of the complement cascade released by the A1 astrocytes (Stevens et al., 2007; Hong et al., 2016; Sekar et al., 2016). It is noteworthy that A1 activation may be initiated by microglia released IL-1β as well as by extracellular Aβ accumulation (Lian et al., 2015; Liddelow and Barres, 2017), both found and here reported in retina of AD patients.

These observations further support the possibility that ocular biomarkers could be used for early detection of AD associated neurodegeneration. It should be noted, however, that other ocular pathologies, such as glaucoma shares histopathological hallmarks with AD including increased levels of tau protein and microglial activation (Ramirez et al., 2017). It is therefore desirable that a complete panel of biomarkers able to discriminate between age-related and disease-related retinal changes would be available and could be used as a target for *in vivo* imaging through a retinal scan. However, this will requires the development of both specific AD biomarkers ligands and long working distance high resolution imaging techniques, in order to achieve a non-invasive and inexpensive diagnosis of AD through the retinal scan.

## Data Availability

The datasets generated for this study are available on request to the corresponding author.

## Ethics Statement

Human Subject Research: The studies involving human participants were reviewed and approved by the Ethics Committees Fondazione Santa Lucia IRCCS. Written informed consent for participation was not required for this study in accordance with the national legislation and the institutional requirements.

## Author Contributions

AG, NP, and FO designed, carried out, and analyzed the immunofluorescence experiments on protein aggregates and neurodegeneration. MR and RP designed, carried out, and analyzed the immunofluorescence experiments on astrocytes and microglia. MG designed, carried out, and analyzed the VCS and spinning disks acquisition. TS and AG designed, performed, and analyzed the FISH experiments. SD wrote the manuscript with the help of RP, GR, DR, and CL. SD conceived the project.

## Conflict of Interest Statement

MG is currently employed at CrestOptics. This study received funding from the JointLab between Istituto Italiano di Tecnologia and CrestOptics. The remaining authors declare that the research was conducted in the absence of any commercial or financial relationships that could be construed as a potential conflict of interest.
